# Natural genetic engineering: intelligence & design in evolution?

**DOI:** 10.1186/2042-5783-1-11

**Published:** 2011-10-31

**Authors:** David W Ussery

**Affiliations:** 1 Center for Biological Sequence Analysis, Department of Systems Biology, The Technical University of Denmark, Kgs. Lyngby, 2800, Denmark

## Abstract

There are many things that I like about James Shapiro's new book "Evolution: A View from the 21st Century" (FT Press Science, 2011). He begins the book by saying that it is the creation of novelty, and not selection, that is important in the history of life. In the presence of heritable traits that vary, selection results in the evolution of a population towards an optimal composition of those traits. But selection can only act on changes - and where does this variation come from? Historically, the creation of novelty has been assumed to be the result of random chance or accident. And yet, organisms seem 'designed'. When one examines the data from sequenced genomes, the changes appear NOT to be random or accidental, but one observes that whole chunks of the genome come and go. These 'chunks' often contain functional units, encoding sets of genes that together can perform some specific function. Shapiro argues that what we see in genomes is 'Natural Genetic Engineering', or designed evolution: "Thinking about genomes from an informatics perspective, it is apparent that systems engineering is a better metaphor for the evolutionary process than the conventional view of evolution as a select-biased random walk through limitless space of possible DNA configurations" (page 6).

In this review, I will have a look at four topics: **1.) **why I think genomics is not the whole story; **2.) **my own perspective of *E. coli *genomics, and how I think it relates to this book**; 3.) **a brief discussion on "Intelligence, Design, and Evolution"; and finally, **4.) **a section "in defense of the central dogma".

## Genomics is not enough

Merely knowing the DNA sequence of the genome does not give the full picture; knowledge of biological systems can provide a more robust explanation. The emergence of novel functions often comes from the 'retention, duplication, and diversification of evolutionary inventions' (page 133 [1]). For example, the evolution of a novel system of motility has been found in *Myxococcus xanthus*, apparently resulting from an ancient duplication and then diversification of genes originally involved in sporulation [2]. As Shapiro warns the reader, there are many parts of the book that are technical. The case is built up for an analogy between the genome and a read/write storage system, which can, in a sense, program itself. This is in contrast to the traditional view that DNA is for storage only, with occasional change through small incremental mutations. The technical details are meant to present the current views of the subject, and in some ways this section feels a bit like browsing through some of the recent publications in a journal such as Genome Research, with details about genome rearrangements and genomic islands and large regions coming and going from chromosomes. At the time of writing, I read an editorial in Science magazine (7 October, 2011), with the title "Genomics is not enough", about how "Genes and their products almost never act alone, but in networks with other genes and proteins and in context of the environment." This is what is meant by Systems Biology - the subject of evolution as addressed in Shapiro's book. Although Shapiro seems to have difficulty in describing the exact function of a gene, from a bacterial perspective, the concept of a 'gene' is both useful and easy to define - it is just a piece of DNA that encodes a functional RNA. Some of these RNAs encode proteins, others form stable RNAs, and together these products form a complex, which has a particular function. The cell can be thought of as a collection of biopolymer complexes, which can form a cognitive system. The cell can 'think' in that it processes signals from the environment and then acts on those signals, in some cases rearranging the genome to accommodate a new and different environment. This is the whole point of Shapiro's book - that the cells can 'design' their own evolution!

## Systems Biology of E. coli

**Figure 1:**
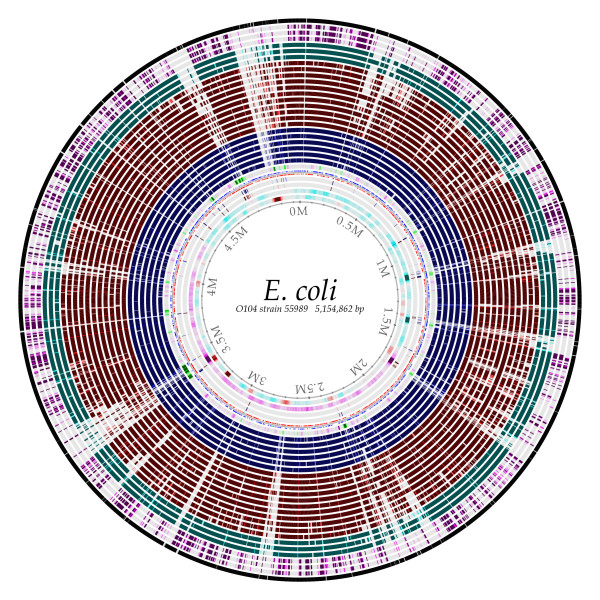
**A BLAST atlas **[8]**of *Escherichia coli *O104 strain 55989, compared to five other *E. coli *O104 isolates (inner blue circles), eleven other pathogenic *E. coli *strains (middle red circles), three commensal, non-pathogenic *E. coli *strains (turquoise circles), and three *H. influenza *genomes (outer violet circles)**. The outermost black circle represents the reference *E. coli *55989 compared to itself; since only the protein encoded genes are compared, the gaps shown are due to intergenic and non-protein encoding regions of the chromosome. A full legend, listing all the strains compared is available as Additional File 1.

I work with bacterial genomics, so it is natural for me to think about genome evolution in terms of phages and genomic islands coming and going from a bacterial chromosome. It is a cruel world out there for the poor bacteria - there are more than 10 phages (bacterial viruses) for every one bacterial cell! Many bacterial genomic islands contain sets of genes which can be thought of as encoding a 'system' - for example, a set of proteins which together can form a type three secretion complex, allowing the bacteria to attach to a eukaryotic cell and inject a protein (such as a toxin, in a bad case) into the cell. Figure 1 shows the conservation of a reference genome (this is one of the sequenced chromosomes from an *E. coli *O104 strain) compared to other O104 genomes from the outbreak in Germany this summer (solid blue circles). Each circle represents comparison to a different genome. I have added other pathogenic E. coli genomes (red circles) and non-pathogenic, commensal *E. coli *genomes (turquoise). The three outermost purple circles represent matches to three *Haemophilus influenza *genomes, which is a distant cousin of *E. coli*. The entire reference genome is more than 5 million bp long, so this means that one pixel wide in the innermost circle represents a bit more than 2000 bp, or roughly 2 genes. The white gaps that can be seen scattered throughout are regions with tens or hundreds of genes, are present in the reference genome, but missing in the various other strains. There are large solid colored regions that are quite similar in all *E. coli *genomes. These conserved regions become smaller and thinner in the outer lanes representing three *H. influenza *genomes. This particular serotype of *E. coli *(O104) was known, but relatively obscure until a major outbreak occurred a few months ago in Germany. Historically, strains of this serotype have not been pathogenic, although they are resistant to many antibiotics. The outbreak strain has an additional virus (bacteriophage) inserted in the genome, near the top of the figure - this virus contains two Shiga toxin genes, which are a source of food-poisoning. From my perspective, this is a good example of the types of changes that Shapiro talks about - in this case the insertion of a new 'system' the phage genes which contain toxins. In experiments with *E. coli *grown continuously in culture over many years, convergent evolution can be seen in the genome (and the results are as expected, affecting DNA topology [3].

## Intelligence, Design, and Evolution

Most people don't have problems with the evolution of bacteria, although in the US it seems that many have problems with the evolution of humans. Shapiro points out that there is a fear of teleology within biology, in part due to historical friction between science and religion - many scientists are simply not comfortable with the idea that an organism has a 'purpose' or is 'designed'. "A shift from thinking about gradual selection of localized random changes to sudden genome restructuring by sensor network-influenced cell systems is a major conceptual challenge.... The emphasis is systemic rather than atomistic and information-based rather than stochastic." (pages 145-146) Inspired by Jim Watson's Molecular Biology of the Gene, the title for first chapter in my textbook is "Life Obeys the Laws of Chemistry and Physics", and in my lectures for my course for the past several years, I've used Stephen Meyers' book, Signature in the Cell [4], as an example of a claim that somehow life is 'special' and cannot be explained by the traditional laws of physics and chemistry - something 'extra' is needed. My point is that Meyers is merely giving us the logical conclusion of a bad analogy [5]. If it is really true that the DNA is only a string of characters, representing some complicated computer program that exists independent of media - then who wrote the program? This analogy holds that DNA is just like a language, made up of letters, and the meaning is not dependent on the physical existence of the letters, but the more abstract ideas that are associated with a given set of letters. I tell my students that, in fact, with DNA, the sequence is important because the particular order of base sequences determines the shape of the DNA helix, and it is the shape that determines function. So in this sense, I wonder whether perhaps Shapiro pushes the analogy too far in the same direction as Meyers in attempting to relate genomic evolution with information science.

## In defense of the central dogma

Yes, it is technically true that the central dogma (DNA makes RNA makes protein) cannot fully explain cellular function, but there is more to the genome than merely the DNA sequence. Shapiro gives a list of genomic functions (DNA compaction, proofreading, replication, *etc*.) that cannot be explained from the central dogma. But I think these functions can be explained from the perspective of the 'sequence hypothesis': the structures of the biopolymers (DNA, RNA, and proteins) are determined by their sequences. Thus, where/how/when a piece of DNA is compacted depends on the particular sequence of nucleotides. Similarly, making sure that mRNAs are in the right place at the right time can be encoded by leader sequences (usually 5' untranslated, 5'UTR).

Having another look at the figure, the inner circles represent DNA structural properties of the reference genome sequence, with the inner-most circle showing the AT content (darker red is AT rich, turquoise is GC rich), followed by GC skew (the bias of the G's towards the leading strand - from this it is easy to see that the origin of replication is in the top right part of the circle, about -45 degrees). The next two lanes are direct and inverted repeats (blue and red, respectively), then the location of the genes is plotted, followed by a prediction of how readily the DNA sequence will be condensed by chromatin proteins - the green regions tend not to be compacted very well, and notice that they correspond to many of the gaps in other genomes. Although for some it could be a useful analogy to think of the genome from an 'informatics' point of view, on the other hand, it is also possible to build up a solid understanding of the functions from a physical/chemical perspective as well. It is possible to have different levels of explanation for the same thing.

Finally, a point where Shapiro and I would agree is that sometimes the recruitment of a single gene, in terms of the right regulator for example, can give a bacterial population the ability to adapt to an ecological niche [6]. There is evidence this has given rise to a new 'species'; I think most would agree that selection plays an obvious role here. (Shapiro makes a somewhat strange claim that "It is important to note that selection has never led to formation of a new species, as Darwin postulated... page 121, but based on what he writes in the rest of the book, I suspect he is here thinking of the need for variations to act on - so in this sense, selection is not technically 'creating' a new species.) We have found clusters of *V. cholera *specific genes that might be responsible for adaption to a particular environment, and to me it seems clear that selection is acting at the genomic level to keep these genomic islands present within a species [7]. Thus, selection is working on natural variation - randomness is still there, in the background, but there is a level of 'jumps' that seem to defy the old adage *Natura non facit saltum*, or 'Nature does not make leaps' - sometimes it does! But this has to be seen in the larger picture of evolved complicated systems and network engineering.

Overall this book is worth the read, although I found that it progressively began to make more sense as a whole after I'd gone through it a couple of times. In my opinion, science needs theories in order to frame and interpret what we see. Shapiro is offering here a glimpse of what the framework of evolution might look like in the near future.

## Competing interests

The authors declare that they have no competing interests.

## Authors' contributions

I have read the book, outlined the paper, and written this article, and I approved the final version of the manuscript.

## Supplementary Material

Additional file 1 **Additional figure legend with descriptions of genomes for the BLAST atlas in Figure **1. This contains the full listing of the 23 bacterial genomes used in Figure 1, including the strain names and colours used, as well as descriptions for the other genomic features of the reference strain plotted in the figure, such as percent AT and GC skew. Click here for file
