# Supplementary figures and images for: Natural genetic engineering: intelligence & design in evolution?
Source: Microb Inform Exp. 2011 Oct 31;1:11. doi: 10.1186/2042-5783-1-11 (PMC3372291; doi:10.1186/2042-5783-1-11)

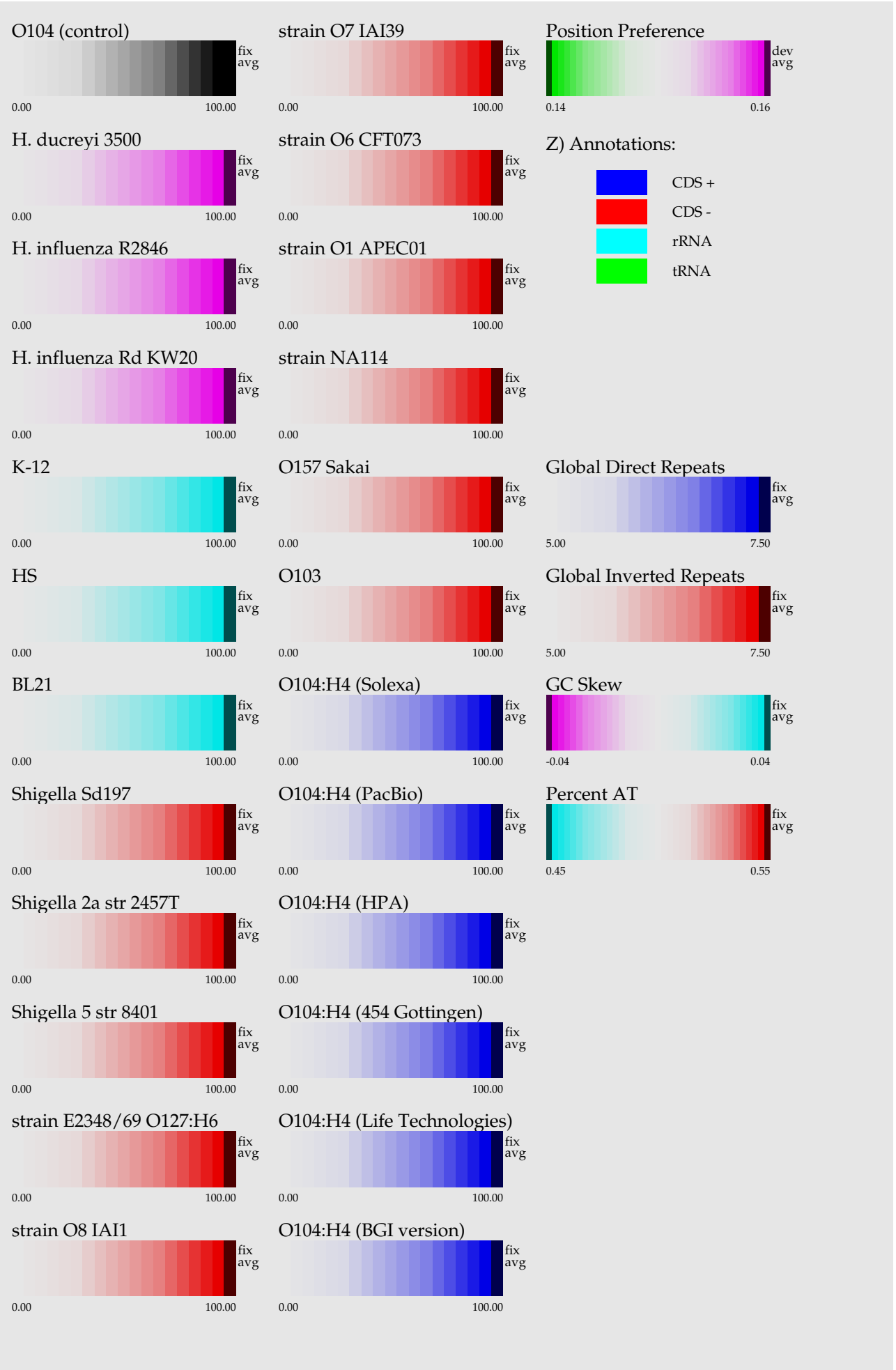

Supplement: Additional file 1 — Additional figure legend with descriptions of genomes for the BLAST atlas in Figure 1. This contains the full listing of the 23 bacterial genomes used in Figure 1, including the strain names and colours used, as well as descriptions for the other genomic features of the reference strain plotted in the figure, such as percent AT and GC skew. [file 2042-5783-1-11-S1.PDF]
